# Familial Interstitial Lung Disease Caused by Mutation of the *STING1* Gene

**DOI:** 10.3389/fped.2020.00543

**Published:** 2020-09-08

**Authors:** Jinying Li, Shuhua An, Zhongdong Du

**Affiliations:** ^1^Department of Pediatrics, Beijing Children's Hospital, Capital Medical University, Beijing, China; ^2^Department of Respiratory, Children's Hospital of Hebei Province, Shijiazhuang, China

**Keywords:** familial interstitial lung disease, transmembrane protein 173 (TMEM173), stimulator of interferon genes (STING), mutation, polyarthralgia

## Abstract

Mutations that affect the *STING1* (TMEM173) gene cause a rare autoinflammatory syndrome, which is known as STING-associated vasculopathy with onset in infancy (SAVI) and which was initially described in 2014 (1). Thus far, only four reports have been conducted regarding families affected with SAVI in the literature. In this article, the clinical, laboratory, and genetic characteristics of two generations (three cases) of SAVI are described. Unlike previously reported cases that were caused by *STING1* mutation, the initial and major clinical manifestations of the mentioned cases are largely identified in the lungs with interstitial lung disease (ILD), and the evidence of typical extrapulmonary symptoms of early-onset systemic inflammation (e.g., cutaneous vasculopathy) were minimal except for the proband, who was diagnosed with arthritis 8 years after onset. In addition, a younger sibling showed no symptoms. Such reports are rarely related to mutations in *STING1*. The proband was examined with bronchoscopy and alveolar lavage to determine the cause. This study emphasizes that, in the clinical assessment of interstitial pneumonia in children, the possibility of *STING1* mutation should be considered, especially in patients with arthritis in addition.

## Introduction

In childhood, ILD refers to a heterogeneous group of rare pulmonary conditions with complex etiologies. Some gene mutations have been found to be related to systemic autoinflammatory diseases that cause ILD. In 2014, a novel autoinflammatory syndrome, a novel type I interferonopathy attributed to mutations in *STING1*, termed STING-associated vasculopathy with onset in infancy (SAVI), was initially reported. SAVI is characterized by early onset systemic inflammation related to cutaneous vasculitis and tissue damage in addition to interstitial lung disease. Because fewer relevant cases have been reported and the associated phenotype is highly variable, more studies should be conducted to delve into the disease. Furthermore, the phenotype of ILD and rheumatoid factor positive (RF+) polyarticular arthritis is reported even less frequently. Accordingly, this study reports three familial cases of this new phenotype, which is expected to expand the spectrum of this disease.

## Case Presentation

### Methods

#### Ethical Approval

The study was approved by the institutional review board of the Children's Hospital of Hebei Province, and informed consent was obtained. Consent for minors was signed by their parents.

#### Patient Recruitment and Clinical Characteristics

The index case is a boy, age 9 when enrolled, who presented with a persistent cough after physical activity over the course of more than 8 years and joint pain for 4 months. He was the first full-term child born via vaginal delivery of unrelated parents of Chinese ethnic background with a body weight of 4.6 kg at birth. Shortly after birth, the patient showed fatigability after light activity and activity-induced tachypnea and cough. A chest X-ray and lung computed tomography (CT) showed bronchial pneumonia with interstitial changes, and percutaneous oxygen saturation was 95% in room air. Severe febrile attacks were rare, and the patient did not seek treatment regularly. At the time of enrollment, the patient weighed 32 kg and was experiencing no failure to thrive (FTT), no obvious abnormality in growth and development, and no tissue lesions involving the skin.

Four months before admission, the patient developed migratory polyarthralgia, affecting the bilateral fingers and toes, wrists, knees, and other joints with swelling at the proximal joints of the middle finger. After the administration of the musk rheumatism capsule (main ingredients include Sichuan aconite, scorpion, earth dragon, black bean, beehive, and artificial musk), the patient's symptoms were alleviated. However, the pain worsened 3 days before admission. Laboratory tests showed a rheumatoid factor (RF) of 47.9 IU/ml (normal: 14 IU/ml) and erythrocyte sedimentation rate (ESR) of 75 mm/h (prior to admission).

The proband's only sibling is a 4 year-old boy, who is asymptomatic; however, CT of the chest showed interstitial changes.

The proband's father presented with shortness of breath post-exercise at the age of 18 after an episode of illness. Chest radiographs and CT showed persistent pathological changes of interstitial pneumonia. At the time of enrollment, the patient's percutaneous oxygen saturation was 93% in room air.

None of the patients needed supplemental oxygen or experienced tachypnea in daily life, but the father experienced mild dyspnea during heavy physical activity.

The proband's mother is healthy. The proband's father has a sister from the same parents who has no similar symptoms. The proband's grandmother and grandfather were both healthy.

#### Laboratory and Other Investigation

The proband and his father and brother underwent chest X-ray or CT. In addition, the proband's pulmonary function and indicators of systemic inflammation were examined.

#### Whole Genome Sequencing and Sanger Sequencing

Targeted exome sequencing (TES) was performed on DNA from peripheral blood cells. Genomic DNA was fragmented, ligated with paired-end adaptors, amplified, and purified. A total of 6,110 gene exons and their 50 bp adjacent introns were captured by a TES Kit (SureSelect Focused Exome, Agilent, USA). A DNA library was established by postcapture amplification and purification and then sequenced on Illumina HiSeq X Ten (Illumina, USA). NextGene V2.3.4 software (Softgenetics, USA) was used for sequencing data alignment to the human genome reference (hg19) and variant calling. The mean read depth was 134.01 ×, but reads reached up to 200 × for 96.194% of the target sequences. Meanwhile, annotation information, including conserved nucleotides and amino acids; prediction of biological functions; frequency in healthy population (1000 Genomes, gnomAD, dbSNP database, and local specific databases); and data from HGMD, Clinvar, and OMIM, was added in NextGene V2.3.4, using self-constructed scripts in our lab.

Variants of pathogenicity were chosen according to standards and guidelines for the interpretation of sequence variants published by ACMG in 2015 with HGVS nomenclature. We used NM_198282.3 as the reference sequence for *STING1* gene analysis. Potentially pathogenic mutations were verified using Sanger sequencing with forward primer 5′-GGACTCTATCGTTACAGGCTGAGG-3′, reverse primer 5′-GCTCCATAGCCCCTTCTGACTCT-3′, and a product length of 414 bp.

## Results

### Laboratory, Imaging, and Pulmonary Function Tests

**Figure 1:**
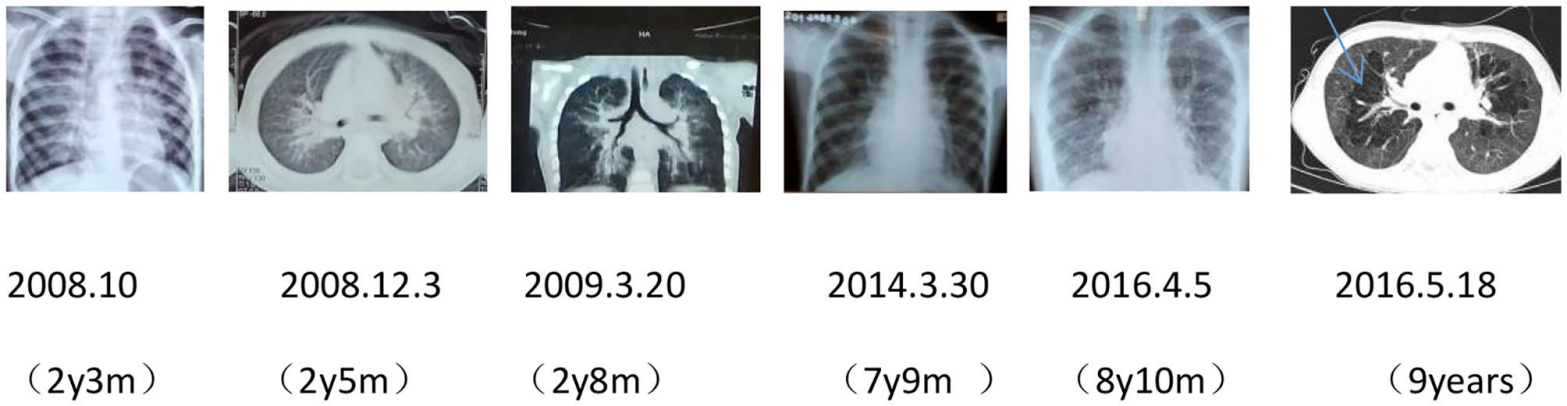
Dynamic chest imaging of the proband (Date of birth: July 3, 2006) suggests gradually worsening interstitial pneumonia and cystic changes and emphysema in the lobular center of the lung on May 18, 2016.

After birth, chest X-ray or CT of the proband showed progressive pulmonary interstitial fibrosis. In addition, there were multiple cystic changes and emphysema in the lobular center of the lung (Figure 1).

**Figure 2:**
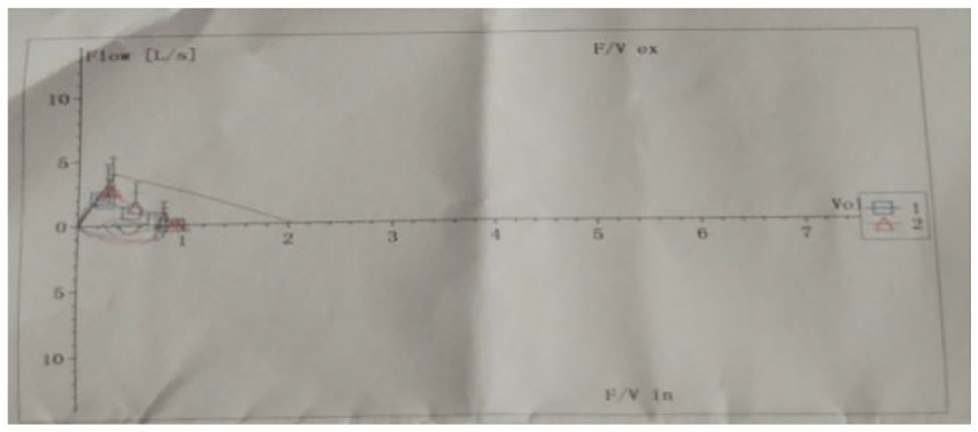
Changes in lung function of the index case show mixed ventilation dysfunction.

**Figure 3:**
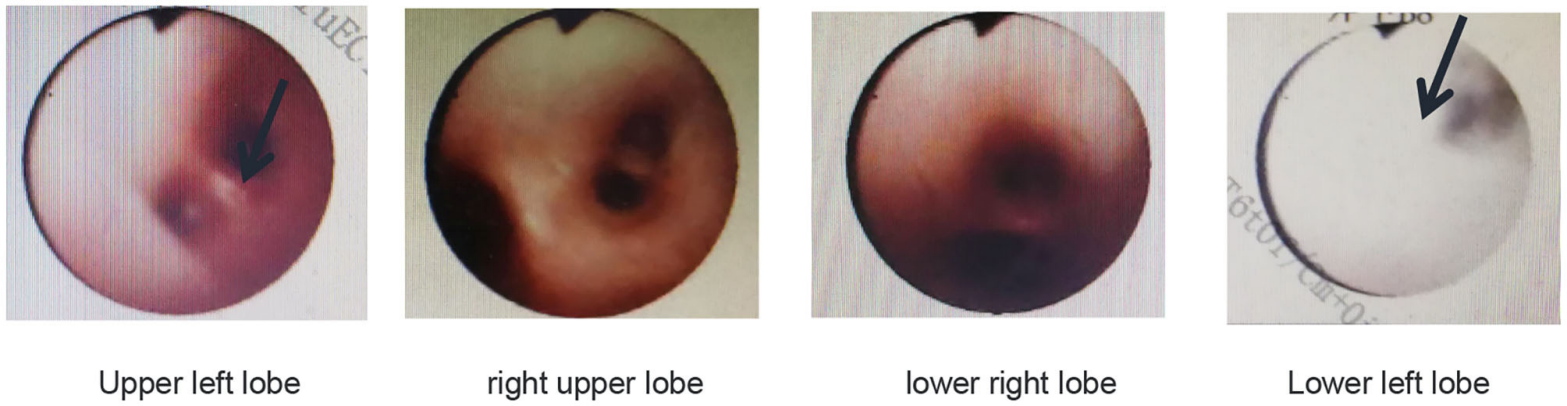
Bronchoscopic images of the index case show that the tracheal mucosa is somewhat hyperemic, coarse, and pale.

**Figure 4:**
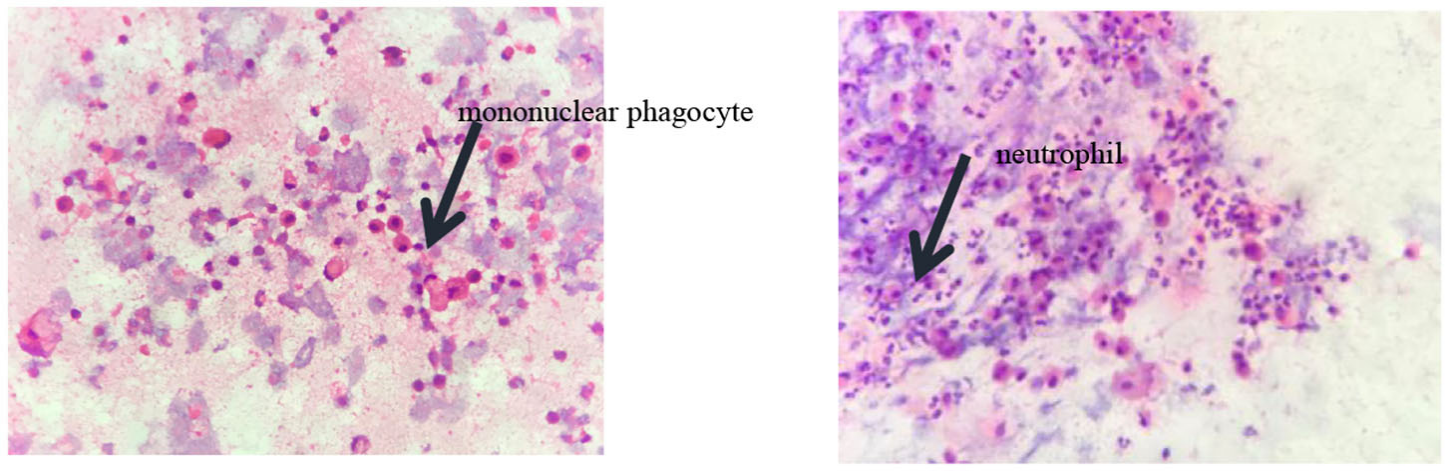
The cytological classification of alveolar lavage fluid suggests that the proportion of neutrophils was 79% and mononuclear phagocytes accounted for 21%.

Pulmonary function tests indicated mixed ventilation dysfunction (Figure 2). Bronchoscopic images of the index case have a coarse and pale appearance. Bronchoscopy found hyperemic tracheal mucosa as shown in the high-magnification smear (Figure 3). Cell classification of bronchoalveolar lavage fluid (BALF) showed 79% neutrophils and 21% mononuclear phagocytes (Figure 4). The high-magnification smear showed red blood cell +, white blood cell ++, epithelial cell +, gram-positive diplococcus+, and gram-negative diplococcus +. Bacterial cultures of alveolar lavage fluid showed normal respiratory flora.

**Figure 5:**
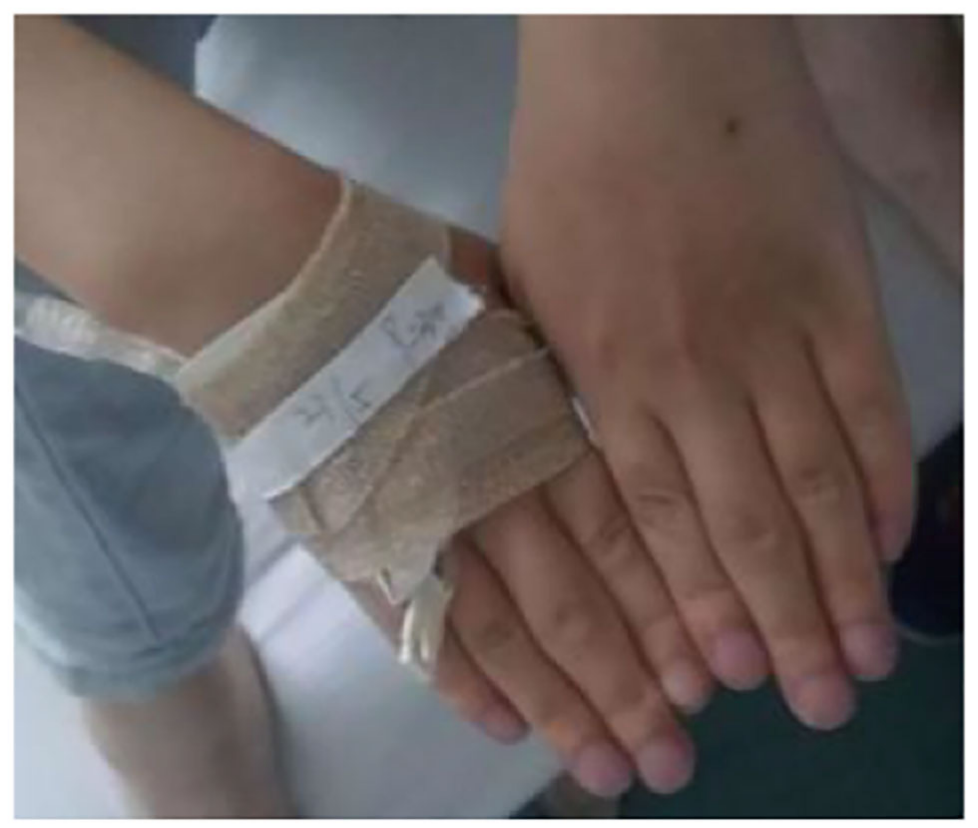
Changes in the hand joints of the index case. The patient has cyanotic, clubbed fingers, and the proximal joints of the middle finger are swollen.

Laboratory investigations revealed a normal white blood cell count in a routine blood examination (WBC 8.7 NE 4.27^*^10^∧^9/L LY4.01^*^10^∧^9/L). During hospitalization, the child developed fever, and the blood routine was reviewed; it showed WBC 12.3^*^10^∧^9/L NE 5.79^*^10^∧^9/L LY 5.83^*^10^∧^9/L systemic inflammation with elevations of C-reactive protein (CRP), high-sensitivity CRP (hs-CRP), and ESR levels (CRP of 39.5 mg/L, hs-CRP of 22.73 mg/L, and ESR of 41 mm/h upon reexamination after admission). Blood CD4+ T was normal. CD8+ T was 38.4% (normal: 20–35%). The ratio of CD4+ to CD8+ cells in the lymphocytes was normal. Total B cells (CD19+) were 18.6% (normal: 5–18%), which was slightly elevated, and natural killer (NK 1.7%) lymphocytes decreased. Hypergammaglobulinemia of IgA and normal IgM and IgG were detected. All autoantibodies were negative (anti-PO, anti-SSA, anti-CENPB, anti-AnuA, anti-AHA, anti-dsDNA, anti-Sm, anti-snRNP, anti–SSB, anti-Jo1, and anti-SCL-70). Indicators of vasculitis (p-ANCA, C-ANCA, MPO, and PR3) were all negative except for p-ANCA. Anticyclic citrullinated peptide antibody was 10.33 RU/ml (normal: 0–25 RU/ml) (Figure 5 Rheumatic changes). C3 was in the normal range, and C4 was elevated to 0.62 g/L (reference range: 0.18–0.4 g/L). No abnormalities were found in procalcitonin (pct 0.076 ug/L; reference range: 0–0.5 ug/L).

A bone marrow puncture showed increased myelocytes and late promyelocytes (14.5%, 17%). Liver, renal, and thyroid functions were normal.

**Figure 6:**
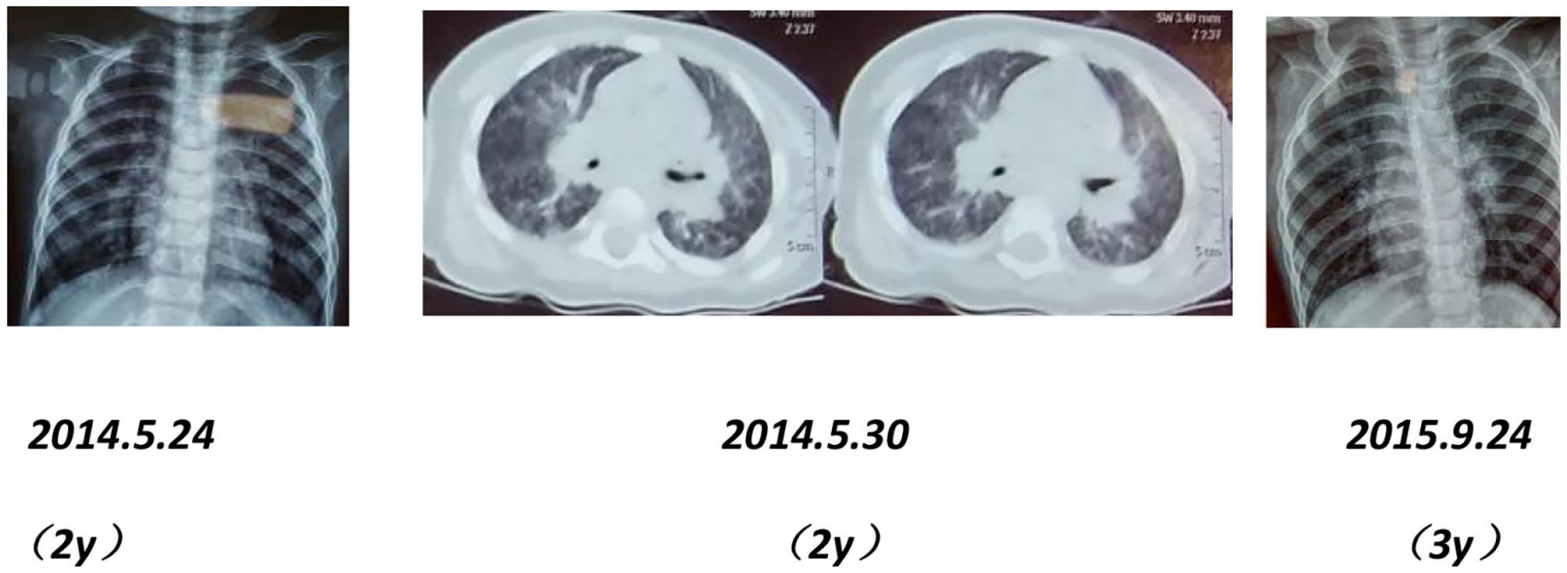
Chest X-rays and CT scans of the proband's younger brother show interstitial pulmonary inflammation.

**Figure 7:**
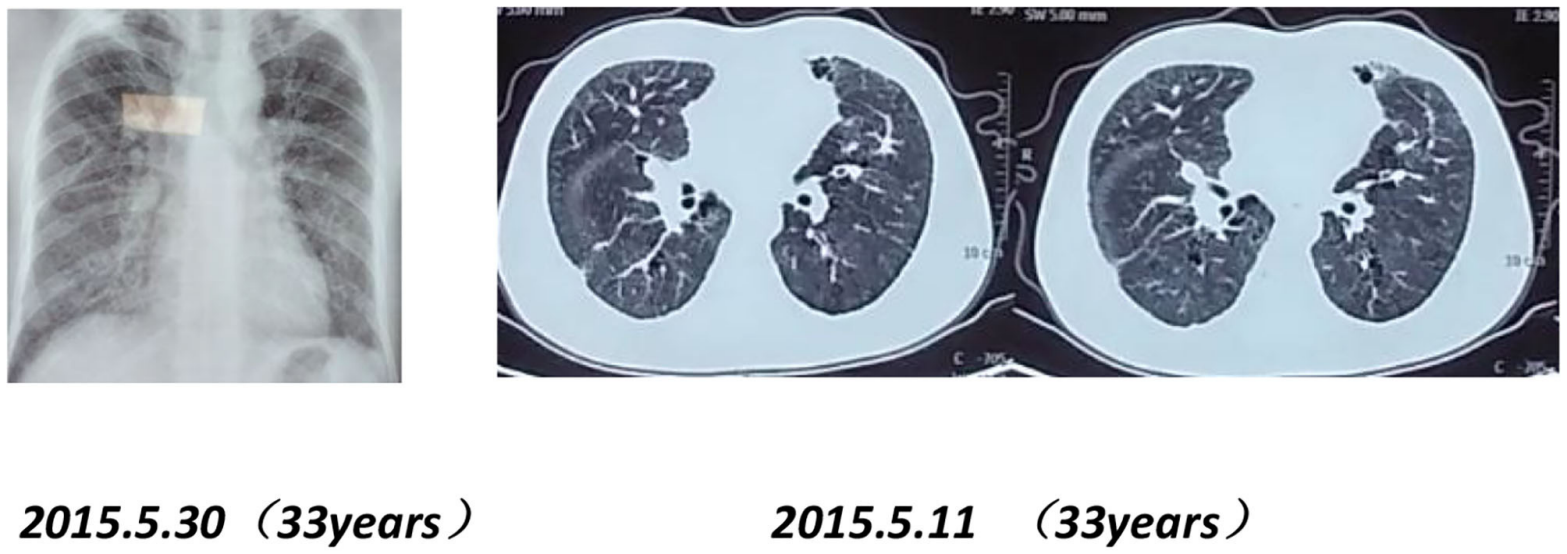
Chest X-rays and CT scans of the proband's father (33 years old) show interstitial pulmonary inflammation.

Chest imaging of the proband's brother and father showed interstitial changes (Figures 6, 7).

### Mutation Analysis

Whole genome sequencing of samples from the index patient was performed, and genetic analysis by Sanger sequencing confirmed a heterozygous mutation (c.842G>A p.Arg281Gln substitution of arginine to glutamine) in the exon 7 of *STING1* (NM_198282.3), e.g., the gene that encodes the stimulator of interferon genes, consistent with SAVI.

**Figure 8:**
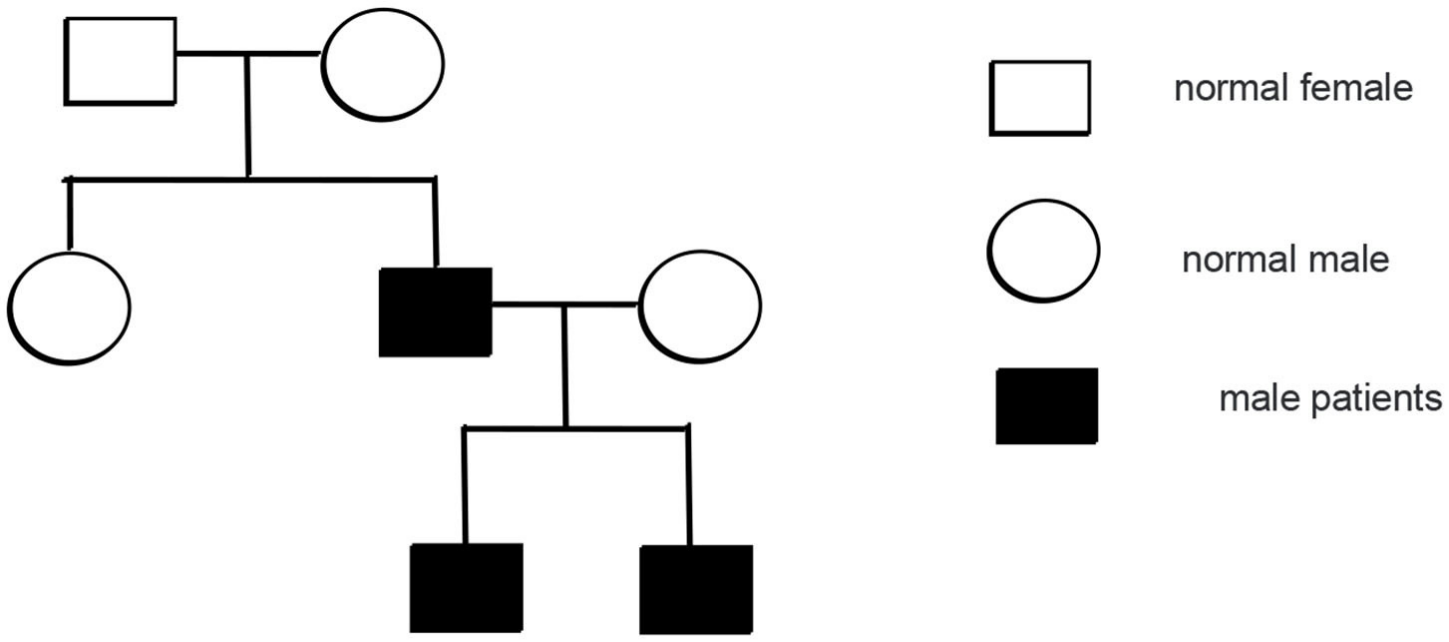
The pedigree of the patients with mutations in TMEM173.

**Figure 9:**
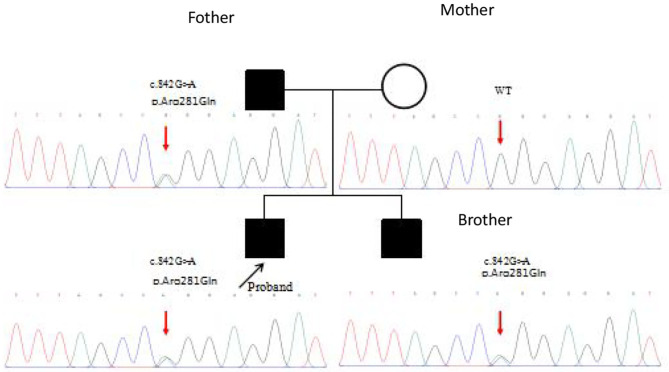
First-generation gene sequencing results.

Mutation analysis revealed that the father and younger brother of the proband both carry the same p.Arg281Gln variant (Figure 7), and his mother is a WT, so the mutation was inherited from the father. The family pedigree is shown in Figures 8, 9.

The proband's aunt, grandmother, and grandfather are healthy, and no DNA samples were available.

### Diagnosis

The diagnosis of SAVI was made due to the presence of interstitial lung disease, systemic inflammation, and genetic analysis results.

### Treatment

After 13 days of non-glucocorticoid therapy for phlegm (acetylcysteine was used for atomization) and 7 days of anti-infection (cefathiamidine) treatment—on the seventh day of hospitalization the patient experienced fever and increased leukocytes in the blood—the patient's symptoms improved, but mild shortness of breath was still present when physically active. However, no oxygen therapy was required.

The proband's younger brother showed no signs of discomfort, but their father could not tolerate heavy physical activity; none of them received treatment.

So far, we have followed the family for more than 3 years. There was no significant change in the proband and his brother with no signs of hypoxia in their normal activity, but his father died of sudden respiratory failure in the third year, which was unanticipated.

## Discussion

Pediatric ILD is a heterogeneous group of rare pulmonary diseases presenting with chronic respiratory pathologies, including inflammatory and fibrotic changes. ILD detected in infancy is often related to genetic mutations (2). At present, mutations on *STING1* and other novel genes, such as COPA, MIM612374, or MIM601924, have been shown to cause systemic autoinflammatory diseases involving the lungs.

*STING1* encodes the STING protein, which is an adaptor molecule linking the sensing of foreign DNA (viral and bacterial) to the production of type 1 IFNs. Its mutations are responsible for type-I IFN overproduction, which has been recently identified as a new cause for interferonopathy, such as SAVI. SAVI is characterized by systemic inflammation (fever, high ESR and CRP levels, IgG and IgA hypergammaglobulinemia) associated with cutaneous vasculitis and tissue damage in addition to interstitial lung disease. To date, 13 identified mutations in the *STING1* gene (V147L, V147M, F153V, N154S, V155M, G166E, C206Y, C206G, G207E, F279L, R281Q, R284G, R284S; https://infevers.umai-montpellier.fr/web/search.php#ancre1768) (1, 3–7) have been described. However, this case study is the first report of *STING1* point mutation (c.842G>A p.Arg281Gln) in Chinese patients.

Lung involvement is reported in up to 90% of previous cases of SAVI (8). In our report, the proband and his brother were found to have imaging changes in interstitial pneumonia starting in infancy, and their father had dyspnea after physical activity from the age of 18 and had the same changes in imaging. In the study by Liu et al. (1), the authors found that five of six patients had evidence of interstitial lung disease on CT though three of them had no respiratory symptoms. STING protein was expressed in bronchial epithelium, alveolar macrophages, and alveolar type II pneumocytes (1). STING mutation results in constitutive activation of the STING–interferon pathway and upregulated type 1 IFN level, resulting in an inflammatory vaso-occlusive process as well as pulmonary lesions, possibly through the activation of alveolar macrophages or pneumocytes (9). In addition, lung toxicity has been reported in patients with multiple sclerosis treated with exogenous type 1 IFN (10), which is consistent with the specific lung pathology seen in SAVI. In this report, usually, they have no fever or other symptoms of infection though the proband was admitted with severe pulmonary fibrosis and no obvious signs of infection. The fiber-optic bronchoscopy suggested the presence of inflammation in the mucosa, but bacterial cultures and cytological classification of a high-magnification smear of alveolar lavage fluid did not support infection although they were consistent with non-infectious inflammation-induced tissue damage, and the BALF showed neutrophilic alveolitis (the percentage of neutrophils in the cell classification was 79%). This change was considered to be caused by mutations in *STING1* that led to a constitutive production of high levels of type I IFNs without infectious triggers (1).

Our results show that all patients carried the same c.842G>A (p.Arg281Gln) mutation as previously reported by Melki in a 7 year-old, white, European ethnicity girl in 2016. The authors used a luciferase-based IFN-β transcription reporter assay and detected robust IFN-β activation *in vitro* with p.Arg281Gln substitution in the *STING1* gene. The variant led to a constitutively active *STING*, and the type I IFN signature was positive in peripheral blood, which was responsible for the disabling autoinflammation in the patient. The authors also confirmed that this variant lies within a novel functional cluster in STING (6).

Common laboratory features of SAVI include raised IgG and IgA and positive autoantibodies—not all of which were observed in our patients. In the index case, IgA was raised, but IgM and IgG were normal, and autoantibodies were negative. SAVI is characterized by systemic inflammation with elevations of CRP, hs-CRP, and ESR levels, which were also characteristic of our patients.

As one of the type I interferonopathies, SAVI has clinical manifestations that are variable even within familial cluster. A literature review reveals that the age of clinical presentation is typically in infancy between birth and 6 months (17/26) (Table 1). Clinical onset in teenage years is rare (3/26) (11). Initial presentations include tachypnea (12), chronic cough (13), telangiectatic erythema, fever and pustular rash, failure to thrive, ischemic acral lesions (14), and even partially necrotizing lesions (15). Among previously described cases, lung involvement occurs in up to 79% of cases (19/24) and usually starts in the neonate period. Not all patients were symptomatic, and some interstitial lung diseases were identified by imaging (16). Our youngest patient was in accordance with this phenotype and was not overtly symptomatic. Respiratory manifestations range from wheezing to tachypnea, chronic cough, and so on (Table 1).

**Table 1:** Clinical features of the patients with mutations in TMEM173 in previously reported patients.

**Number**	**Literature**	**Patient**	**Sex**	**Time of onset**	**Ethnicity/Origin**	**Initial presentation**	**Mutation**
1	[1]	1	Female	Neonatal	French-Canadian	Tachypnea, erythematous tip of nose and cheeks at 4 months of age	N154S
2		2	Male	8 weeks	American/Caucasian	Telangiectatic erythema	V155M
3		3	Female	6 weeks	Turkish	Fever and pustular rash	N154S
4		4	Male	1 week	Hispanic	Fever, ulcers on toes and fingers, generalized rash and pustules on face	V147L
5		5	Female	Neonatal	American/Caucasian	Tachypnea in perinatal period, pustular lesions on the heels and toes since the age of 6 month	N154S
6		6	Male	3 days	American/Caucasian	Swollen fingers	N154S
7	[11]	1	Male (I-4)	Adulthood	French	Underweight	V155M
8		2	Male (II-5)	Teenage years	French	Failure to thrive	V155M
9		3	Male (II-6)	Teenage years	French	Failure to thrive	V155M
10		4	Female (III-2)	1 year	French	Malar rash	V155M
11	[3]		Male	8 weeks		Cutaneous lesions	V147M
12	[13]	1	Male	the first weeks of life	Japanese/Northern European	chronic cough and skin lesions	V155M
13	[15]	1	Female	6 years	***Greek***	Violaceous Papules or purple patches	G166E
14		2	Male	3 years	***Greek***	Violaceous Papules or purple patches	G166E
15		3	Female	2 months	***Greek***	Violaceous Papules or purple patches	G166E
16		4	Female	12 years	***Greek***	Violaceous Papules or purple patches	G166E
17		5	Female	10 years	***Greek***	Violaceous Papules or purple patches	G166E
18	[8]	1	Case 1 Male	1 year	Algerian	Failure to thrive	V155M
19		2	Case 2 Female	20	Algerian	ILD	V155M
20		3	Case 3 Male	5 month	Caribbean	Failure to thrive and an acute viral respiratory infection	V155M
21	[12]		Male	5 weeks	Hazars	Fever, tachypnea	V155M
22	[6]	1	Female	3 months	European	Feeding difficulties and respiratory distress	R281Q
23		2	Female	from birth	European	Failure to thrive widespread livedo reticularis	R284G
24		3	Male	Shortly after birth	European	Fixed erythema	C206Y
25	[16]		Male	6 months	Turkish	Failure to thrive recurrent cough	N154S
26	[14]		Male	Infancy	Turkish	Tachypnea acral necrosis	N154S

However, genotype–phenotype correlations may vary. Compared with this disease as previously reported and the same mutation reported by Melki et al. (6), the symptoms of the patients in our study have significant deviations. The proband and his brother showed a rather mild clinical presentation, and even in the lung CT, the fibrosis was obvious, but their father's breathing was more pronounced during physical activity. The patient in the Melki report presented with feeding difficulties, telangiectatic skin lesions, and damage of liver function. In our cases, the initial and main presentations were interstitial pneumonia; they had few extrapulmonary manifestations and no skin lesions. Considering that other variations in the *STING1* gene may result in a decrease in the expression or function of the haplotype, resulting in a mild clinical phenotype, we further checked the exome sequencing of variants INFAR1 and 2, JAK-STAT, TBK1, and IRF3 to see if the variants were affecting the pathway, and the results were negative.

The other interesting finding is the arthritis of the proband. It is increasingly recognized that type 1 interferonopathies can manifest as musculoskeletal disease (17). A recent study performed DNA sequencing in 100 patients diagnosed with juvenile idiopathic arthritis (JIA) with analysis of all coding exons and flanking introns, including *STING1*. They found that these JIA patients had a relatively frequent incidence of autoinflammatory syndromes (18). In the SAVI literature, arthritis and arthralgia have previously been described as minor features [heterozygous mutation of c.463G> A; p.Val155Met (17)], but for mutation of c.842G>A (p.Arg281Gln) in *STING1*, this is the first report. Type I interferonopathies are a clinically heterogenic group of diseases with a constitutive activation of the type I interferon generate pathway that might present as atypical, severe, early onset rheumatic diseases with which interstitial lung diseases are common (19). It is suggested that the differential diagnosis of such clinical cases should include type I interferonopathies (20), such as SAVI.

Currently, therapeutic options for SAVI are limited, and traditional immunosuppressive medications and biologic therapies have disappointing efficacy (16). No standard immunosuppressive treatment approach is able to control disease progression (21).

Fremond et al. (22) described the efficacy of a selective oral JAK1/2 inhibitor, ruxolitinib, in three children aged between 5 and 12 years with *STING1*-activating mutations; two of them carried a p.V155M mutation, and the other carried a p.V147M mutation. The authors found that JAK inhibition had a marked positive effect on all aspects of the symptoms in all three children, including major improvement in pulmonary function, and was also well-tolerated. However, in another study, the author reported a patient with SAVI (p.ser102pro and p.phe279leu, two variants of *STING1*) who was treated with another Janus kinase inhibitor, tofacitinib, and the patients' skin lesions improved but the pulmonary defects remained unchanged (7). In another report, involving treatment with a JAK1/2 inhibitor, ruxolitinib, in a patient with severe pulmonary involvement of SAVI (c.842G>A p.Arg281Gln mutation in TMEM173), at 18 months of therapy, a CT scan revealed a worsening of the interstitial disease (21). JAK inhibition may be worth considering as a therapeutic approach for some subtypes of SAVI.

Our patients were not treated with a Jak1/2 inhibitor due to limited conditions. On admission, the index required short-term anti-infective treatment and oxygen intake during bronchiolitis, but with no need for ventilator support, he was treated with no immune-modifying agents.

Other therapies for SAVI include TBK1 antagonists, and ER exit-blocking agents may be an option (23, 24). Further study needs to be done to get its effect on SAVI.

From published reports, we can see that some cases of SAVI, especially in patients with severe lung involvement, the prognosis is poor, and in our report, the proband's father died at 38 years old of sudden respiratory failure. Therefore, it is emphasized that such diseases should be treated early even in asymptomatic patients.

Limitations of this study include that we have not been able to perform a cytokine dosage in the patients' plasma or sera.

In summary, we describe three individuals of two generations from the same family with SAVI. It is the first report of this specific variant causing familial SAVI in China. The initial and major clinical manifestations are largely identified in the lungs with ILD, and extrapulmonary symptoms were minimal except for the proband diagnosed with arthritis 8 years after onset. This phenotype has been reported rarely.

## Data Availability Statement

This article contains previously unpublished data. The name of the repository and accession number(s) are not available.

## Ethics Statement

Written informed consent was obtained from the individual(s) and/or minor(s)′ legal guardian/next of kin for the publication of any potentially identifiable images or data included in this article.

## Author Contributions

JL wrote the manuscript under the direction of SA and ZD and his classmate modified the language. We thank them for their efforts. All the authors designed the study and analyzed experimental results together.

## Conflict of Interest

The authors declare that the research was conducted in the absence of any commercial or financial relationships that could be construed as a potential conflict of interest.
